# Supplementary material for: Urinary Long Non-Coding RNA Levels as Biomarkers of Lupus Nephritis
Source: Int J Mol Sci. 2023 Jul 22;24(14):11813. doi: 10.3390/ijms241411813 (PMC10380660; doi:10.3390/ijms241411813)
Supplement: Supplementary file 1 [file ijms-24-11813-s001.zip › ijms-2485637-supplementary.pdf]

Supplementary Table S1. Correlation between urinary lncRNA levels and clinical parameters*

lncRNA targets	MEG3	ANRIL	lnc-MGC	MALAT	CASC2	TUG1
eGFR	r=0.129, p=0.5	r=0.175, p=0.4	r=0.465, p=0.011	r=0.111, p=0.3	r=-0.360, p=0.001	r=0.706, p=0.01
proteinuria	r=-0.423, p=0.018	r=-0.483, p=0.008	r=0.197, p=0.3	r=-0.096, p=0.4	r=-0.025, p=0.8	r=-0.510, p=0.09
SLEDAI	r=-0.383, p=0.034	r=-0.334, p=0.08	r=0.064, p=0.7	r=-0.060, p=0.6	r=-0.138, p=0.2	r=-0.053, p=0.0
anti-ds DNA titre	r =-0.146, p=0.4	r=-0.077, p=0.7	r=0.245, p=0.2	r=-0.011, p=0.9	r=-0.232, p=0.033	r=-0.182, p=0.6
serum C3 level	r=-0.107, p=0.5	r=0.024, p=0.9	r=-0.273, p =0.3	r=0.235, p=0.038	r=0.041, p=0.7	r=-0.053, p=0.9
serum C4 level	r=0.057, p=0.8	r=-0.106, p=0.6	r=-0.093, p=0.6	r=0.134, p=0.2	r=0.089, p=0.4	r=-0.051, p=0.9
kidney pathology						
activity index	r=-0.311, p=0.089	r=-0.142, p=0.5	r=-0.053, p=0.8	r=-0.321, p=0.004	r=-0.217, p=0.056	r=-0.051, p=0.9
chronicity index	r=-0.161, p=0.4	r=-0.286, p=0.13	r=-0.281, p=0.14	r=-0.062, p=0.6	r=-0.096, p=0.4	r=-0.276, p=0.4
glomerulosclerosis	r=-0.015, p=0.9	r=-0.178, p=0.4	r=-0.170, p=0.4	r=-0.119, p=0.3	r=-0.061, p=0.6	r=-0.257, p=0.4
interstitial fibrosis	r=-0.197, p=0.3	r=-0.356, p=0.06	r=-0.189, p=0.3	r=-0.078, p=0.5	r=-0.049, p=0.7	r=-0.276, p=0.4

*Data were analyzed by the Spearman's correlation coefficient.
